# Neutrophil extracellular traps (NETs) are increased in the alveolar spaces of patients with ventilator-associated pneumonia

**DOI:** 10.1186/s13054-018-2290-8

**Published:** 2018-12-27

**Authors:** Carmen Mikacenic, Richard Moore, Victoria Dmyterko, T. Eoin West, William A. Altemeier, W. Conrad Liles, Christian Lood

**Affiliations:** 1 Division of Pulmonary and Critical Care Medicine, Department of Medicine, University of Washington, 325 Ninth Avenue, Box 359640, Seattle, WA 98104 USA; 2 Division of Rheumatology, Department of Medicine, University of Washington, 750 Republican Street, Rm. E563, Box 358060, Seattle, WA 98109 USA; 3 Division of Allergy and Infectious Diseases, Department of Medicine, University of Washington, 1959 NE Pacific Street; HSB RR-511, Box 356420, Seattle, WA 98195 USA; 4 Center for Lung Biology, 850 Republican Street., Rm. S384, Box 358052, Seattle, WA 98109 USA

**Keywords:** Neutrophil extracellular traps (NETs), bronchoalveolar lavage, alveoli, acute respiratory distress syndrome, ventilator-associated pneumonia, cell-free DNA, peroxidase, calprotectin

## Abstract

**Background:**

Neutrophils release neutrophil extracellular traps (NETs) in response to invading pathogens. Although NETs play an important role in host defense against microbial pathogens, they have also been shown to play a contributing mechanistic role in pathologic inflammation in the absence of infection. Although a role for NETs in bacterial pneumonia and acute respiratory distress syndrome (ARDS) is emerging, a comprehensive evaluation of NETs in the alveolar space of critically ill patients has yet to be reported. In this study, we evaluated whether markers of NET formation in mechanically ventilated patients are associated with ventilator-associated pneumonia (VAP).

**Methods:**

We collected bronchoalveolar lavage fluid from 100 critically ill patients undergoing bronchoscopy for clinically suspected VAP. Subjects were categorized by the absence or presence of VAP and further stratified by ARDS status. NETs (myeloperoxidase (MPO)-DNA complexes) and the NET-associated markers peroxidase activity and cell-free DNA were analyzed by enzyme-linked immunosorbent assay and colorimetric assays, respectively. Quantitative polymerase chain reaction of nuclear and mitochondrial DNA was used to determine the origin of the extruded DNA. Interleukin (IL)-8 and calprotectin were assayed as measures of alveolar inflammation and neutrophil activation. Correlations between NETs and markers of neutrophil activation were determined using Spearman’s correlation. We tested for associations with VAP and bacterial burden by logistic and linear regression, respectively, using log_10_-transformed NETs.

**Results:**

MPO-DNA concentrations were highly correlated with other measures of NET formation in the alveolar space, including cell-free DNA and peroxidase activity (*r* = 0.95 and *r* = 0.87, *p* < 0.0001, respectively). Alveolar concentrations of MPO-DNA were higher in subjects with VAP and ARDS compared with those with ARDS alone (*p* < 0.0001), and higher MPO-DNA was associated with increased odds of VAP (odds ratio 3.03, *p* < 0.0001). In addition, NET concentrations were associated with bacterial burden (*p* < 0.0001) and local alveolar inflammation as measured by IL-8 (*r* = 0.89, *p* < 0.0001).

**Conclusions:**

Alveolar NETs measured by MPO-DNA complex are associated with VAP, and markers of NETosis are associated with local inflammation and bacterial burden in the lung. These results suggest that NETs contribute to inflammatory responses involved in the pathogenesis of VAP.

**Electronic supplementary material:**

The online version of this article (10.1186/s13054-018-2290-8) contains supplementary material, which is available to authorized users.

## Background

Mechanical ventilation in critically ill adults can be complicated by ventilator-associated pneumonia (VAP). VAP is associated with mortality greater than 40% in some studies [1, 2]. Mechanical ventilation may also contribute to the pathogenesis of acute respiratory distress syndrome (ARDS) [3, 4]. In patients with ARDS, the development of VAP may be associated with higher risk of death [5].

One of the main contributors to pathological inflammation in both ARDS and VAP are neutrophils. While neutrophils play a protective role against invading pathogens, unrestrained inflammation may lead to tissue injury in the lung [6, 7]. One of the mechanisms by which neutrophils could promote both bacterial killing and contribute to tissue injury is through release of extracellular traps [8–10] . These neutrophil extracellular traps (NETs) are characterized by extrusion of chromatin bound to cytosolic and granular contents of the cells. NETs are composed of DNA complexed with myeloperoxidase (MPO) or citrullinated histones [9, 10]. Surrogate markers of NET formation include peroxidase activity and cell-free DNA (cf-DNA) levels [11–13]. Although NET-derived DNA is primarily of nuclear origin, we recently demonstrated that mitochondrial extrusion could occur concomitantly with NETosis, with released oxidized mitochondrial DNA being highly inflammatory [14, 15]. Thus, NET formation may be a key factor in balancing protective versus harmful inflammation.

In murine models of lung injury, NETs develop in response to a variety of infectious stimuli and contribute to lung injury [16–19]. In humans, plasma NET levels were higher in patients with pneumonia-associated ARDS or transfusion-associated ARDS than in subjects without ARDS [18, 19]. Extracellular histones, likely derived from dying cells, are elevated in bronchoalveolar lavage fluid (BALF) and the plasma of subjects with ARDS compared with healthy controls [20]. Nevertheless, a thorough examination of NETs in the alveolar space collected by bronchoalveolar lavage in critically ill mechanically ventilated patients has not been reported to date.

In this study, we sought to evaluate the levels of traditional markers and novel markers of NETosis in BALF from mechanically ventilated ICU patients. We hypothesized that markers of NETosis would be associated with microbially confirmed VAP.

## Methods

### Subjects

BALF specimens were collected from mechanically ventilated patients undergoing bronchoscopy for suspected VAP (*n* = 100) in intensive care units at Harborview Medical Center (Seattle, WA). VAP was defined according to the IDSA guidelines by a quantitative bacterial culture of > 10,000 CFU/ml in the BALF [21]. ARDS was defined according to the Berlin criteria as respiratory failure with acute onset in the setting of a known clinical insult, bilateral opacities on chest imaging, and hypoxemia with PaO_2_/F_i_O_2_ ≤ 300 not fully explained by cardiac failure on the day of bronchoscopy [22]. The onset of ARDS was defined as the day of the first qualifying P/F ratio combined with an appropriate chest x-ray. Subjects were included if they were mechanically ventilated in the medical, surgical, or neurologic intensive care units and bronchoscopy was performed if the patient had new radiologic abnormalities, leukocytosis, purulent secretions, or fever. Subjects were excluded if they were less than 18 years of age, pregnant, prisoners, had a known diagnosis of HIV, previous bone marrow or solid organ transplant, or were admitted for severe burns or with metastatic cancer. The characteristics of this study population have been previously described [23]. This part of the study was approved by the University of Washington’s Human Subjects Committee and samples were obtained under a waiver of consent.

BALF was also obtained from healthy subjects undergoing research bronchoscopy as previously described [24]. This part of the study was also approved by the University of Washington’s Human Subjects Committee and samples were obtained after written consent.

### Sample processing

The BALF specimen was filtered through a 70-μm cell strainer and then centrifuged at 300 × g for 5 min for critically ill subjects and for 10 mins at 4 °C for healthy subjects. The supernatant was collected as BALF and frozen at –80 °C. All samples were thawed only once to perform the following studies.

### Analysis of soluble markers of neutrophil activation and inflammation

NETs were analyzed as described previously [14, 25]. Briefly, anti-human MPO (clone 4A4, AbD Serotec) and anti-DNA-HRP (Cell Death Detection ELISA Kit, Roche) were used as capture and detection antibodies. The NET-related markers peroxidase activity and cf-DNA were measured by colorimetric assays [14, 25]. Interleukin (IL)-8 concentrations were measured by immunoassay (Mesoscale Discovery, Rockville, MD). S100A8/A9 (calprotectin) levels were analyzed by enzyme-linked immunosorbent assay (ELISA; R&D Systems). Samples that fell below the lower limit of detection were assigned that value.

Bronchoalveolar lavage mitochondrial (mt) and genomic (g) DNA content was analyzed by measuring the copy number of two mitochondrial genes (*MT*-*RNR2*, *MT*-*TL1*) and two genomic genes (*B2M*, *RNA18SN5*). Briefly, 8 μl of BALF was mixed with 10 μl of *power*sybr Green PCR Master Mix (Applied Biosystems, Foster City, CA, USA) and 2 μl of forward (12.5 μm) and reverse (12.5 μm) primers for *MT-RNR2*, *MT-TL1*, *B2M*, or *RNA18SN5* at a final volume of 20 μl (Additional file 1: Table S1). As a standard curve, synthesized oligonucleotides of the target sequence were used (Additional file 2: Table S2). Each quantitative real-time polymerase chain reaction (PCR) was run on a steponeplus™ Real-Time PCR System (Applied Biosystems, Foster City, CA, USA). Samples were denatured at 95 °C for 15 min prior to 40 cycles of amplification (95 °C for 15 s, 60 °C for 60 s). Sequence copy numbers were calculated by comparing C_T_ values of samples and a standard curve.

### Statistics

Statistics were performed on untransformed NET concentrations using nonparametric tests. For comparisons across multiple groups we performed the Kruskal-Wallis test and post-test Dunn’s to adjust for multiple comparisons. Correlations were performed using the Spearman’s rank-order correlation. For regression analyses, NET concentrations were log_10_-transformed. We adjusted for age and gender given literature that supports both age and sex in affecting innate immune response, particularly in the lung [1, 2]. We further adjusted for ARDS as it could affect both NET formation and risk for VAP but would not necessarily be on the causal pathway between the two. Analyses were performed using GraphPad Prism (La Jolla, CA) and Stata 14 (College Station, Texas).

## Results

### Subject characteristics

All mechanically ventilated subjects underwent bronchoscopy for clinical suspicion of VAP (*n* = 100). On average, subjects were 52 (±18) years of age and predominantly male (*n* = 79, 80%). Subjects were relatively evenly distributed amongst those without ARDS or VAP (*n* = 25), those with ARDS but without VAP (*n* = 19), those without ARDS but with VAP (*n* = 30), and those with both ARDS and VAP (*n* = 26). The average length of mechanical ventilation prior to bronchoscopy did not differ greatly by clinical condition and ranged from 4.3 to 7.8 days (Table 1). Of the subjects meeting ARDS criteria, the majority did so prior to the day of bronchoscopy with the exception of three subjects who met ARDS criteria on the day of bronchoscopy. The subjects overall had a high severity of illness measured by Acute Physiology and Chronic Health Evaluation (APACHE) III score (mean score across groups 75.8–87.7) and high mortality (rate across groups 21–36%). ARDS subjects were predominantly from surgical rather than medical ICUs, and there was a high proportion of trauma-related ARDS (Table 1). The four healthy subjects ranged in age from 18 to 22 and 75% were female.

**Table 1:** Patient demographics by clinical condition

Demographics	No ARDS or VAP *n* = 25	ARDS/no VAP *n* = 19	VAP/no ARDS *n* = 30	VAP and ARDS *n* = 26
Age, mean (SD)	49 (18)	53 (16)	57 (16)	48 (21)
Male sex, *n* (%)	22 (92)	16 (84)	19 (63)	22 (85)
Race, *n*				
Caucasian	17	13	25	20
African American	2	2	1	2
Native American	3	2	2	2
Asian	0	1	1	1
Other/mixed race	2	1	0	1
ICU type, *n*				
Medical	9	2	1	1
Surgical	7	20	18	23
Neurological	8	2	11	2
ARDS risk factor, *n*				
Trauma	7	6	18	20
Aspiration	2	7	1	5
Sepsis	1	6	0	1
Other reason for MV	14	0	11	0
APACHE III, mean (SD)	75.8 (28.7)	87.7 (39.2)	77.5 (22.6)	86.7 (22.7)
Days of MV prior to BAL, mean (SD)	4.3 (5.3)	7.8 (7.2)	5.2 (4.1)	5.8 (5.4)
Mortality, *n* (%)	8 (36)	4 (21)	9 (30)	7 (27)

*APACHE* Acute Physiology and Chronic Health Evaluation, *ARDS* acute respiratory distress syndrome, *BAL* bronchoalveolar lavage, *ICU* intensive care unit, *MV* mechanical ventilation, *SD* standard deviation, *VAP* ventilator-associated pneumonia

### Variability of NETs in critical illness

We first comprehensively characterized NET concentrations (MPO-DNA complexes) and other markers of NETosis (peroxidase and cf-DNA) in BALF from critically ill subjects. NETs were detectable in BALF from all subjects with a median (interquartile range (IQR)) of 223 (40.6–766) U/mL. Of the patients included in the study, 69 subjects were not on antibiotics for 48 h prior to bronchoscopy. Thirty-one subjects were on antibiotics, but these antibiotics had not changed 48 h prior to the bronchoscopy. We compared the level of NETs by MPO-DNA complex in subjects on antibiotics with those not on antibiotics and there was no significant difference (*p* = 0.13). In healthy subjects, NETs were undetectable in all four BALF samples. Further markers of NETosis were not obtained in the healthy subjects. Peroxidase activity (6.40 (1.34–15.5) mU/mL) and cf-DNA (87.74 (20.0–253) ng/mL) varied widely across critically ill subjects. We have previously shown that both nuclear and mitochondrial DNA are present in NETs, with mitochondrial DNA being more proinflammatory [14]. The range of nuclear DNA content by 18S RNA and β-2 microglobulin ranged from 7881 (441–49,257) copies/μL and 7026 (297–39,765) copies/μL, respectively. Mitochondrial DNA content was also highly variable, ranging from 65.3 (25.3–253) copies/μL for 16S RNA and 1229 (333–3863) copies/μL for MT-TL1. There was a strong correlation between NET concentration and peroxidase activity and cf-DNA (*p* < 0.0001; Table 2). However, correlations between content of 18S, β-2 microglobulin, mitochondrial 16S, and MT-TL1 DNA content and NETs were either only weakly associated or independent. These data show that, across a spectrum of mechanically ventilated patients with and without pneumonia or ARDS, NETs and other markers of NETosis were present and highly variable.

**Table 2:** Correlations between markers of NETosis in critically ill patients receiving mechanical ventilation

	MPO-DNA	Peroxidase	cf-DNA	18S	β2M	16S DNA	MT-TL1
MPO-DNA	1.00						
Peroxidase	0.87***	1.00					
cf-DNA	0.95***	0.81***	1.00				
18S	0.06	−0.02	0.08	1.00			
β2M	0.31*	0.14	0.26	0.17	1.00		
16S	0.05	0.02	0.09	0.24	−0.01	1.00	
MT-TL1	0.21	0.13	0.25	0.26	0.36**	0.03	1.00

*p* values are Bonferroni corrected

Values are Spearman’s correlations

*β2M* β-2 microglobulin, *cf-DNA* cell-free DNA, *MPO-DNA* myeloperoxidase DNA

***p < 0.0001, **p < 0.01, *p < 0.05

### NETs and clinical associations with VAP

**Fig. 1:**
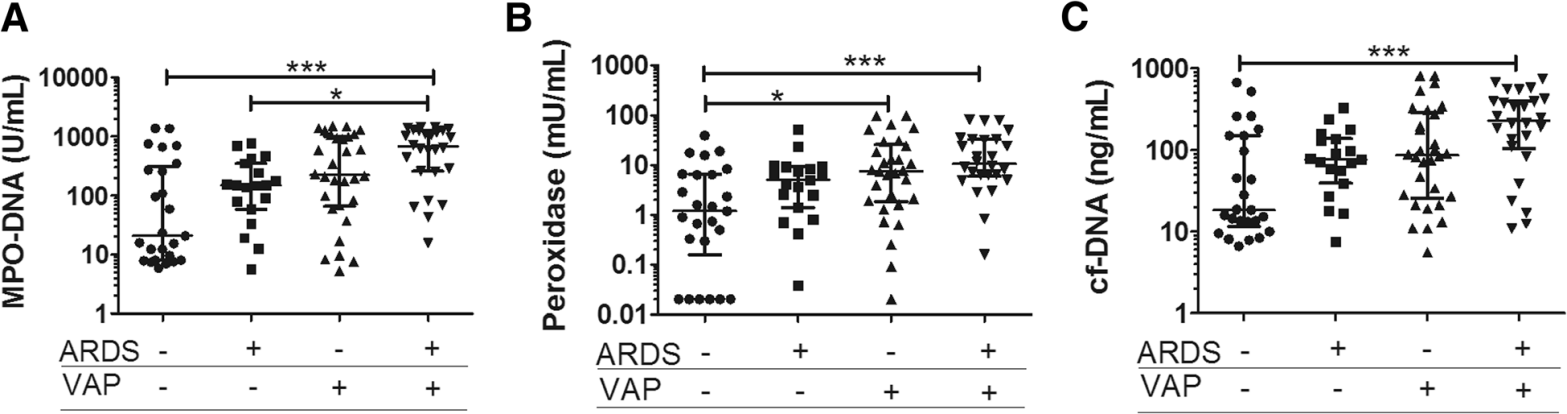
NETs and NETosis markers by clinical group. **a** Myeloperoxidase (MPO)-DNA, **b** peroxidase activity, and **c** cell-free DNA (cf-DNA) are compared in ventilator associated pneumonia (VAP) with and without acute respiratory distress syndrome (ARDS). MPO-DNA is higher in ARDS patients with VAP. *P* values are for Kruskal-Wallis (nonparametric analysis of variance (ANOVA) with post-test Dunn’s to account for multiple pairwise tests). Error bars show the median and interquartile range. ***p < 0.0001, *p < 0.05

We next wanted to identify whether there were correlations between NETs and markers of NETosis and VAP. We compared NET concentrations between subjects with or without VAP further stratified by ARDS status. We found that MPO-DNA complex, peroxidase, and cf-DNA concentrations were significantly different between these groups by Kruskal-Wallis (all *p* < 0.0001; Fig. 1). After adjusting for multiple comparisons, MPO-DNA complexes in subjects with ARDS and VAP were higher than those with ARDS alone (*p* < 0.05; Fig. 1a). We further tested for associations between increased NETs and risk of VAP by logistic regression. For each log_10_ increase in MPO-DNA complex concentration, there was an increased risk of VAP (odds ratio (OR) 3.03, 95% confidence interval (CI) 1.69–5.43; *p* < 0.0001). Multiple logistic regression to adjust for age, gender, and ARDS status showed that increased log_10_ (MPO-DNA) was again associated with VAP (OR 3.75, 95% CI 1.91–7.35; *p* < 0.0001). When we restricted the analysis to patients without VAP, MPO-DNA complexes were not associated with ARDS (OR 2.09, *p* = 0.11). Peroxidase activity and cf-DNA were also associated with increased odds of VAP (Table 3).

**Table 3:** Association between markers of NETosis and ventilator-associated pneumonia (VAP) in critically ill mechanically ventilated patients

Markers of NETosis (log_10_-transformed)	OR (95% CI)	*p* value	Adjusted OR (95% CI)	*p* value
MPO-DNA	3.03 (1.69–5.43)	< 0.0001	3.75 (1.91–7.35)	< 0.0001
Peroxidase	2.57 (1.33–4.95)	0.005	2.89 (1.45–5.75)	0.002
cf-DNA	3.09 (1.50–6.25)	0.002	3.84 (1.69–8.77)	0.001

Adjusted odds ratio (OR) is for multiple logistic regression adjusted for age, gender, and presence/absence of acute respiratory distress syndrome

*cf-DNA* cell-free DNA, *CI* confidence interval, *MPO-DNA* myeloperoxidase DNA

Neither nuclear DNA content by 18S or β-2 microglobulin DNA nor mitochondrial DNA content by 16S and MT-TL1 DNA were statistically different amongst these populations (Additional file 3: Figure S1). Additionally, we did not identify any associations between markers of NETosis and ARDS severity or mortality. These results show that NET concentrations differ amongst subjects with ARDS and/or VAP and that the presence of VAP is strongly associated with NET concentrations.

### NETs, neutrophil activation, and local inflammation

**Fig. 2:**
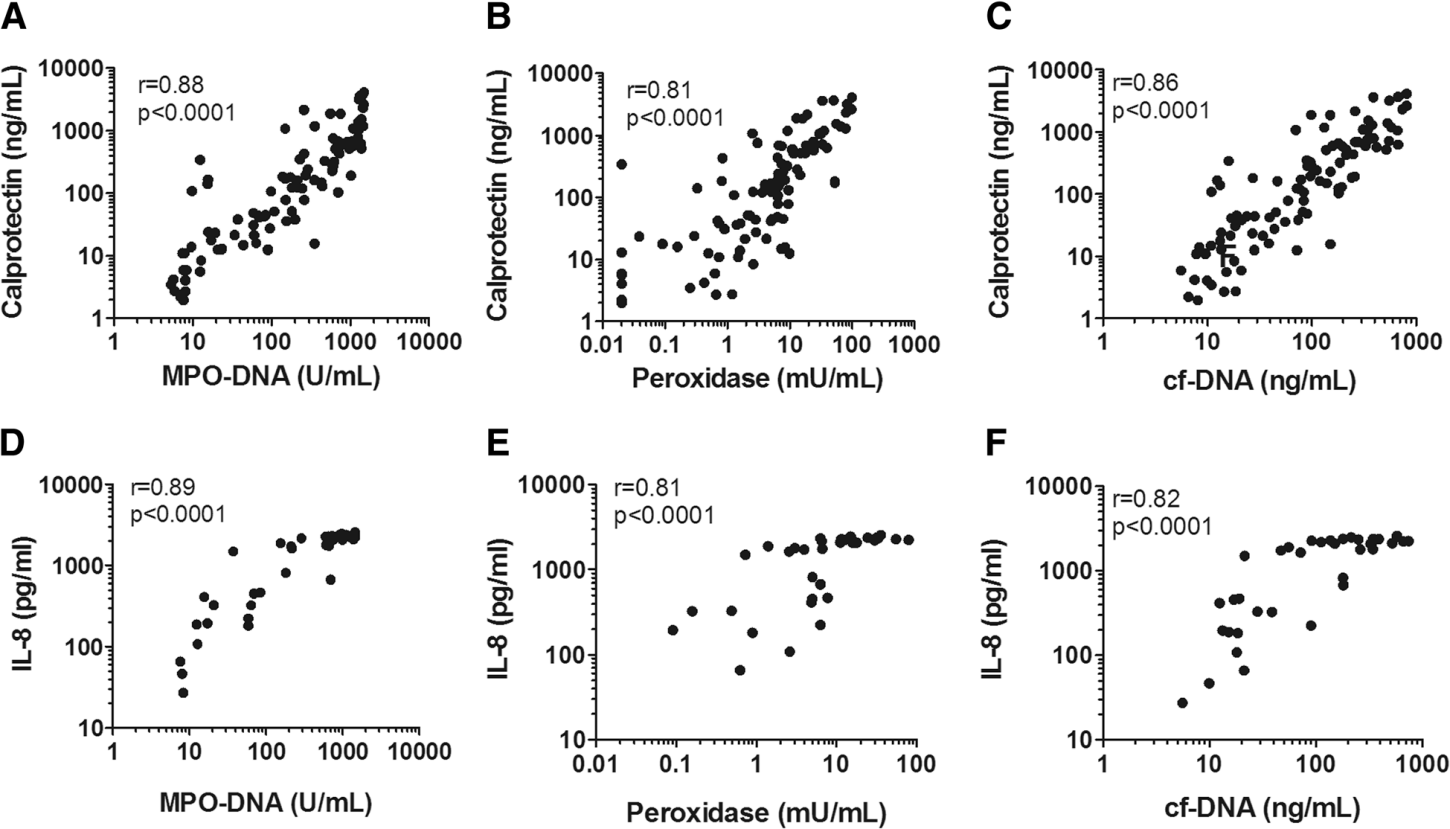
NETs and neutrophil activation. NETs are associated with **a–c** the neutrophil cytosolic protein calprotectin and **d–f** the neutrophil chemoattractant interleukin-8 (IL-8). The correlation was a Spearman’s correlation for nonparametric data. Calprotectin (ug/mL) and IL-8 (pg/mL) were both measured by immunoassay. cf-DNA cell-free DNA, MPO-DNA myeloperoxidase DNA

We subsequently determined whether MPO-DNA and markers of NETosis were associated with other measures of neutrophil activation or local inflammation in the alveolar space. Calprotectin, also known as S100A8/A9, is a cytosolic protein measure of neutrophil activation [26]. The chemokine IL-8 is a known marker of inflammation in ARDS and VAP and a strong neutrophil chemoattractant [27]. We found that both MPO-DNA complex (*r* = 0.88, *p* < 0.0001), peroxidase (*r* = 0.81, *p* < 0.0001), and cf-DNA (*r* = 0.86, *p* < 0.0001) were positively correlated with calprotectin (Fig. 2a–c). There was a similarly strong correlation to levels of IL-8 (Fig. 2d–f). This indicates a strong link between NETosis and local inflammation and neutrophil degranulation.

### NETs and bacterial burden

**Fig. 3:**
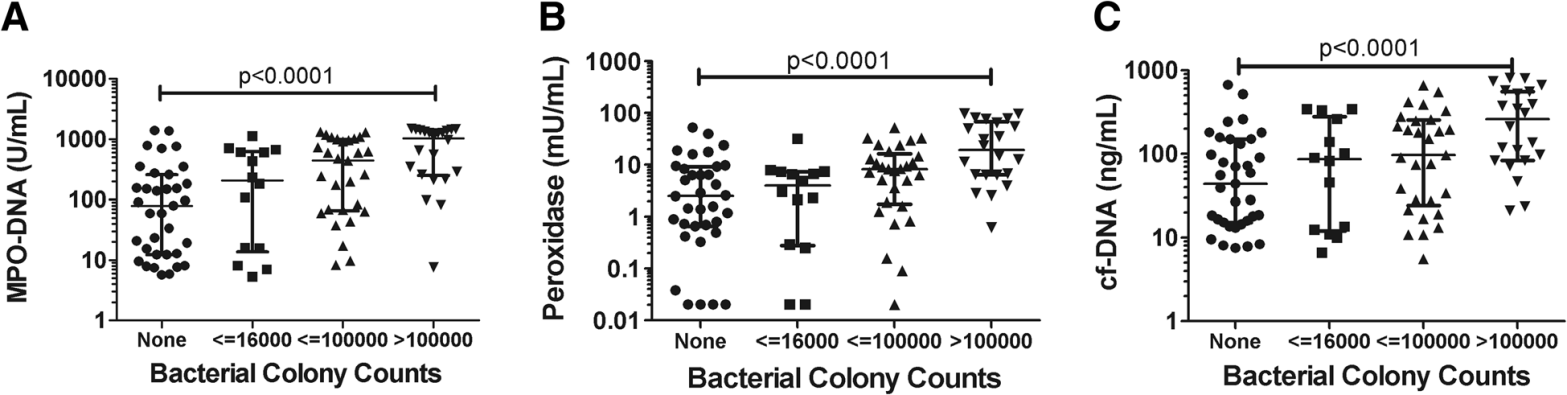
NETs and bacterial burden**.** Bacterial colony counts from quantitative bronchoalveolar lavage cultures are represented by quartile of increasing colony count versus measures of NETs and NETosis. Presented *p* values are from regression analysis with log-transformed **a** myeloperoxidase (MPO)-DNA, **b** peroxidase, or **c** cell-free DNA (cf-DNA) concentrations and quartile of colony count

Quantitative bacterial cultures for each subject provided us with the ability to identify associations between the burden of bacteria present in the alveolar space and concentrations of NETs. With each higher quartile of bacterial content, there was an increase in log-transformed concentration of MPO-DNA complexes (β = 0.30, *p* < 0.0001), peroxidase (β = 0.24, *p* < 0.0001), and cf-DNA concentrations (β = 0.19, *p* < 0.0001; Fig. 3). Because bacterial content is linked to the presence VAP, we wanted to test whether bacterial burden within clinical groups with VAP was still associated with increased NETosis. In subjects with both ARDS and VAP or VAP alone, we dichotomized patients into high and low colony count groups. We found that amongst subjects with both VAP and ARDS, MPO-DNA concentration and peroxidase activity were significantly higher in subjects with higher bacterial colony counts (*p* = 0.03 and *p* = 0.02, respectively; Additional file 4: Figure S2A–C). Similarly, MPO-DNA concentration and cf-DNA were higher with higher bacterial colony counts in subjects with VAP alone (Additional file 4: Figure S2D–F). When we compared concentrations of markers of NETosis and bacterial quality by gram stain in all critically ill subjects, there was no difference in MPO-DNA concentration, peroxidase activity, or cf-DNA concentration (Additional file 5: Figure S3A–C). Taken together, increasing bacterial burden is associated with increased NETosis.

## Discussion

Our study broadly assessed NETosis in the alveolar space of critically ill patients. We found that the most direct measure of NETs, MPO-DNA complexes, is higher in patients with microbially confirmed VAP. Increasing bacterial burden in the alveolar space was also associated with increased NETosis. This suggests that direct bacterial infection strongly drives NETosis and has implications for the future study of both ARDS and bacterial pneumonia. Extrapolation of our results would suggest that NETosis in the alveolar space of patients with direct ARDS due to pneumonia may differ from subjects with indirect ARDS. We could not directly address this in our study due to the limited number of patients with ARDS.

We also found that other markers of NETosis in the alveolar space correlate highly with MPO-DNA complexes. Cell-free DNA and peroxidase activity also differed by clinical condition and were highly correlated with MPO-DNA complexes suggesting they could serve as reasonable surrogates for NETosis. In particular, peroxidase activity was higher in patients with VAP alone compared with patients without VAP or ARDS. This again supports that bacterial infection is a strong driver of the process of NETosis. Consistent with this finding, we determined that MPO-DNA complexes, cell-free DNA, and peroxidase activity all increased with increasing bacterial burden measured by quantitative culture. Because this study measured alveolar concentrations rather than circulating concentrations of NETs, our findings suggest local, intra-alveolar NETosis in the presence of bacterial infection in the lung.

NETosis also correlated with alveolar inflammation as measured by IL-8. IL-8 is a neutrophil chemoattractant and has been shown to be associated with ARDS outcomes and VAP [27–29].

Calprotectin is a dimer of calcium binding proteins S100A8 and S100A9 and is a measure of neutrophil degranulation [30]. In our study, calprotectin also correlated with NETosis in the alveolar space. A previous proteomic study of BALF showed that S100A8 was differentially expressed in ARDS patients with VAP compared with those without VAP [31]. Our study thus provides a strong link between local alveolar inflammation, neutrophil activation, and NETosis.

Our study has several limitations. First, we do not have corresponding plasma samples to correlate the degree of alveolar NETosis with circulating concentrations of NETs. Future studies will address whether circulating concentrations can serve as a surrogate for direct alveolar measurement. Second, subjects included in this study were ventilated for a variable number of days prior to alveolar sampling. This may in part explain why we found stronger associations for patients with VAP because clinical bronchoscopy was performed for this indication. Third, the use of samples obtained from clinically indicated bronchoscopy limits our ability to provide analyses for ARDS since patients had ARDS for a variable number of days prior to alveolar sampling. Finally, because all of our subjects were at risk for VAP with mechanical ventilation for > 48 h, the severity of critical illness is high for all subjects in this study including those without ARDS or VAP. We did measure MPO-DNA complexes from healthy subjects and the amounts were undetectable. This suggests that, at baseline, mechanical ventilation likely induces some degree of neutrophilic inflammation.

Whether NETs serve a protective role in the setting of bacterial infection with VAP and/or play a role in tissue damage remains unclear. Previous studies provide support for NETs playing a role in tissue injury in the lung [16, 18, 32]. There are also dynamic factors that may influence NET formation in the alveolar space. For example, BALF from ARDS patients has recently been shown to promote NET production from neutrophils [33]. In community-acquired pneumonia, higher circulating amounts of cell-free nucleosomes was associated with higher 30-day mortality, showing a link between a marker of NETosis and poor clinical outcome [34]. These studies support our hypothesis that NETs serve as a marker of alveolar inflammation and may contribute to tissue injury and poor outcomes in critical illness.

## Conclusions

Neutrophil-extracellular traps (NETs), measured as MPO-DNA complexes, are present in the alveolar space in critically ill patients. Higher MPO-DNA complexes are associated with VAP, higher bacterial burden, and with other markers of alveolar and neutrophilic inflammation. This study suggests a role for NETs in VAP pathogenesis, and NETs may be considered as a marker of neutrophilic inflammation in the alveolar space.

## Additional files

Additional file 1: **Table S1.** Primer sequences for mitochondrial and genomic genes. (DOCX 13 kb)

Additional file 2: **Table S2.** Synthesized oligonucleotides of target sequences for the standard curve. (DOCX 13 kb)

Additional file 3: **Figure S1.** Nuclear DNA content and mitochondrial DNA content do not differ by clinical group. Nuclear DNA by (A) β-2 microglobulin (*B2M*)] and (B) 18S ribosomal RNA (*RNA18SN5*) and mitochondrial DNA by (C) mitochondrial encoded 16S RNA (*MT-RNR2*) and (D) mitochondrial-encoded tRNA leucine 1 (*MT-TL1*) content was quantified by quantitative PCR. *P* values are for Kruskal-Wallis (nonparametric analysis of variance (ANOVA)). (DOCX 1448 kb)

Additional file 4: **Figure S2.** NETs and bacterial burden in patients with VAP. Bacterial colony counts from quantitative bronchoalveolar lavage cultures are represented by quartile of increasing colony count versus measures of NETs and NETosis. Subjects are dichotomized into high and low colony count groups amongst those with ARDS and VAP (A–C) or VAP alone (D–F). *P* values are for Mann-Whitney nonparametric pairwise tests. Error bars show the median and interquartile range. (DOCX 622 kb)

Additional file 5: **Figure S3.** NETs and bacterial type. NETs are not associated with bacterial type. MPO-DNA, peroxidase, or cell-free DNA concentrations were compared amongst cultures with gram-negative, gram-positive, both forms of organisms, and cultures that only grew oral flora. *P* values are for Kruskal-Wallis nonparametric analysis of variance (ANOVA). Error bars show the median and interquartile range. (DOCX 461 kb)

## Abbreviations

ARDS: Acute respiratory distress syndrome
BALF: Bronchoalveolar lavage fluid
cf-DNA: Cell-free DNA
CI: Confidence interval
IL: Interleukin
MPO: Myeloperoxidase
NET: Neutrophil extracellular trap
OR: Odds ratio
VAP: Ventilator-associated pneumonia
