# Supplementary material for: Neutrophil extracellular traps (NETs) are increased in the alveolar spaces of patients with ventilator-associated pneumonia
Source: Crit Care. 2018 Dec 27;22:358. doi: 10.1186/s13054-018-2290-8 (PMC6307268; doi:10.1186/s13054-018-2290-8)
Supplement: Supplementary file 1 — Table S1. Primer sequences for mitochondrial and genomic genes. (DOCX 13 kb) [file 13054_2018_2290_MOESM1_ESM.docx]

| Gene | Forward primer (5’-3’) | Reverse Primer (5’-3’) |
| --- | --- | --- |
| *MT-RNR2* | CTTTGCAAGGAGAGCCAAAG | GACGGGTGTGCTCTTTTAGC |
| *MT-TL1* | GAACAGGGTTTGTTAAGATGGC | GAGGAATTGAACCTCTGACTGTA |
| *B2M* | TGCTGTCTCCATGTTTGATGTATCT | TCTCTGCTCCCCACCTCTAAGT |
| *RNA18SN5* | ATGGCCGTTCTTAGTTGGTG | ATGCCAGAGTCTCGTTCGTT |

**Additional file 1: Table S1. Primer sequences for mitochondrial and genomic genes**
