# Supplementary material for: Neutrophil extracellular traps (NETs) are increased in the alveolar spaces of patients with ventilator-associated pneumonia
Source: Crit Care. 2018 Dec 27;22:358. doi: 10.1186/s13054-018-2290-8 (PMC6307268; doi:10.1186/s13054-018-2290-8)
Supplement: Supplementary file 2 — Table S2. Synthesized oligonucleotides of target sequences for the standard curve. (DOCX 13 kb) [file 13054_2018_2290_MOESM2_ESM.docx]

**Additional file 2: Table S2. Synthesized oligonucleotides of target sequences for standard curve**

| Gene | Synthesized oligonucleotide (5’-3’) |
| --- | --- |
| *MT-RNR2* | CTTTGCAAGGAGAGCCAAAGCTAAGACCCCCGAAACCAGACGAGCTACCTAAGAACAGCTAAAAGAGCACACCCGTC |
| *MT-TL1* | GAACAGGGTTTGTTAAGATGGCAGAGCCCGGTAATCGCATAAAACTTAAAACTTTACAGTCAGAGGTTCAATTCCTC |
| *B2M* | TGCTGTCTCCATGTTTGATGTATCTGAGCAGGTTGCTCCACAGGTAGCTCTAGGAGGGCTGGCAACTTAGAGGTGGGGAGCAGAGA |
| *RNA18SN5* | ATGGCCGTTCTTAGTTGGTGGAGCGATTTGTCTGGTTAATTCCGATAACGAACGAGACTCTGGCAT |
