# Supplementary material for: Neutrophil extracellular traps (NETs) are increased in the alveolar spaces of patients with ventilator-associated pneumonia
Source: Crit Care. 2018 Dec 27;22:358. doi: 10.1186/s13054-018-2290-8 (PMC6307268; doi:10.1186/s13054-018-2290-8)
Supplement: Supplementary file 3 — Figure S1. Nuclear DNA content and mitochondrial DNA content do not differ by clinical group. Nuclear DNA by (A) β-2 microglobulin (B2M)] and (B) 18S ribosomal RNA (RNA18SN5) and mitochondrial DNA by (C) mitochondrial encoded 16S RNA (MT-RNR2) and (D) mitochondrial-encoded tRNA leucine 1 (MT-TL1) content was quantified by quantitative PCR. P values are for Kruskal-Wallis (nonparametric analysis of variance (ANOVA)). (DOCX 1448 kb) [file 13054_2018_2290_MOESM3_ESM.docx]

Additional file 3: Figure S1.


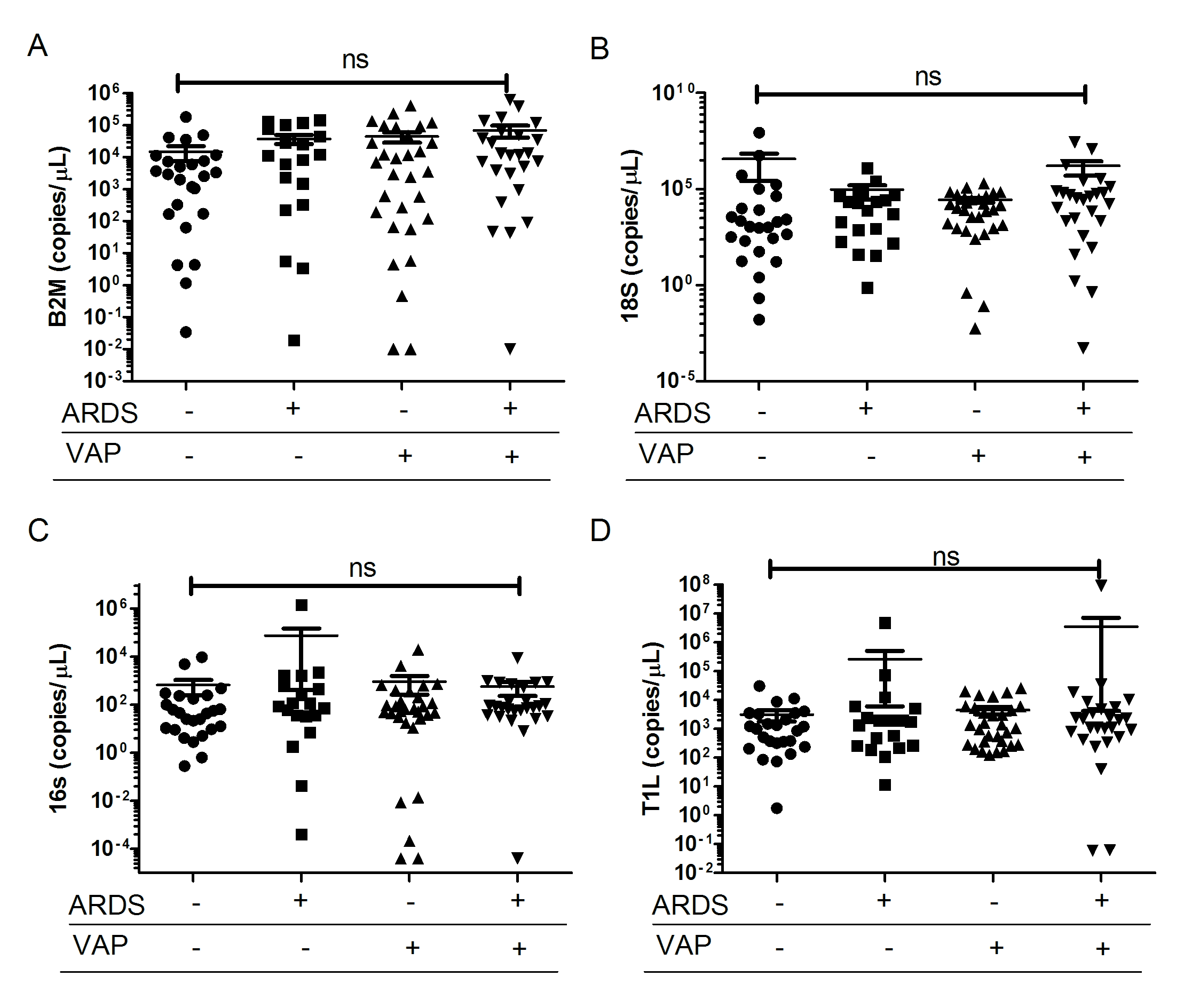


**Additional file 3: Figure S1. Nuclear DNA content and mitochrondrial DNA content do not differ by clinical group**. Nuclear DNA by A) β-2 microglobulin (*B2M*)] and B) 18s ribosomal RNA (*RNA18SN5*) and mitochondrial DNA C) mitochondrial encoded 16s RNA (*MT-RNR2*) and D) mitochondrial-encoded tRNA leucine 1 (*MT-TL1*)] content was quantified by qPCR. P values are for Kruskal-Wallis (non-parametric ANOVA).
