# Supplementary material for: Neutrophil extracellular traps (NETs) are increased in the alveolar spaces of patients with ventilator-associated pneumonia
Source: Crit Care. 2018 Dec 27;22:358. doi: 10.1186/s13054-018-2290-8 (PMC6307268; doi:10.1186/s13054-018-2290-8)
Supplement: Supplementary file 4 — Figure S2. NETs and bacterial burden in patients with VAP. Bacterial colony counts from quantitative bronchoalveolar lavage cultures are represented by quartile of increasing colony count versus measures of NETs and NETosis. Subjects are dichotomized into high and low colony count groups amongst those with ARDS and VAP (A–C) or VAP alone (D–F). P values are for Mann-Whitney nonparametric pairwise tests. Error bars show the median and interquartile range. (DOCX 622 kb) [file 13054_2018_2290_MOESM4_ESM.docx]

**Additional file 4: Figure S2.**

**
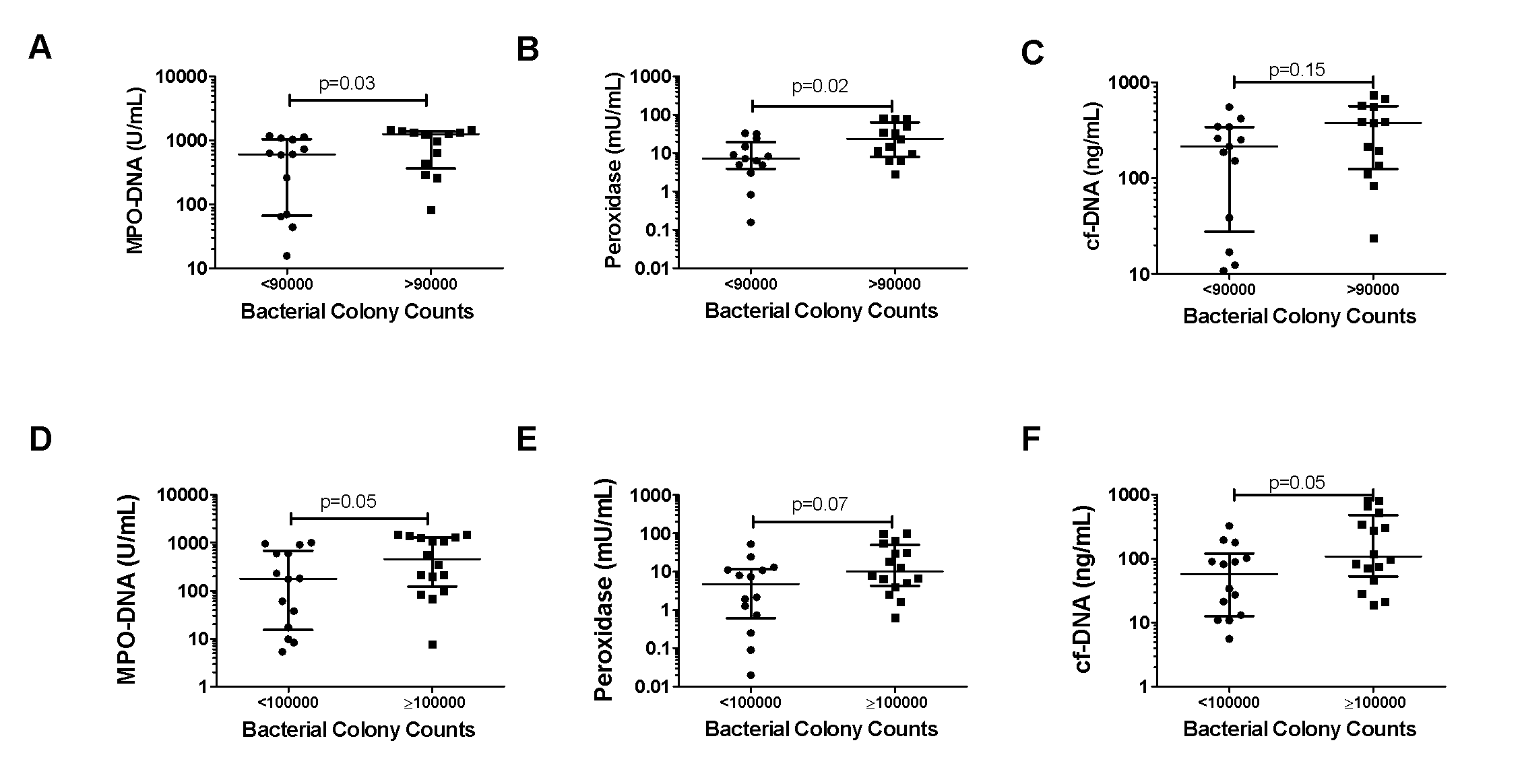
**

**Additional file 4: Figure S2. NETs and bacterial burden in patients with VAP.** Bacterial colony counts from quantitative bronchoalveolar lavage cultures are represented by quartile of increasing colony count versus measures of NETs and NETosis. Subjects are dichotomized into high and low colony count groups amongst those with ARDS and VAP (A,B,C) or VAP alone (D,E,F). P values are for Mann-Whitney non-parametric pairwise tests. Error bars show the median and interquartile range.
