# Supplementary material for: Neutrophil extracellular traps (NETs) are increased in the alveolar spaces of patients with ventilator-associated pneumonia
Source: Crit Care. 2018 Dec 27;22:358. doi: 10.1186/s13054-018-2290-8 (PMC6307268; doi:10.1186/s13054-018-2290-8)
Supplement: Supplementary file 5 — Figure S3. NETs and bacterial type. NETs are not associated with bacterial type. MPO-DNA, peroxidase, or cell-free DNA concentrations were compared amongst cultures with gram-negative, gram-positive, both forms of organisms, and cultures that only grew oral flora. P values are for Kruskal-Wallis nonparametric analysis of variance (ANOVA). Error bars show the median and interquartile range. (DOCX 461 kb) [file 13054_2018_2290_MOESM5_ESM.docx]

**Additional file 5: Figure S3.**

**
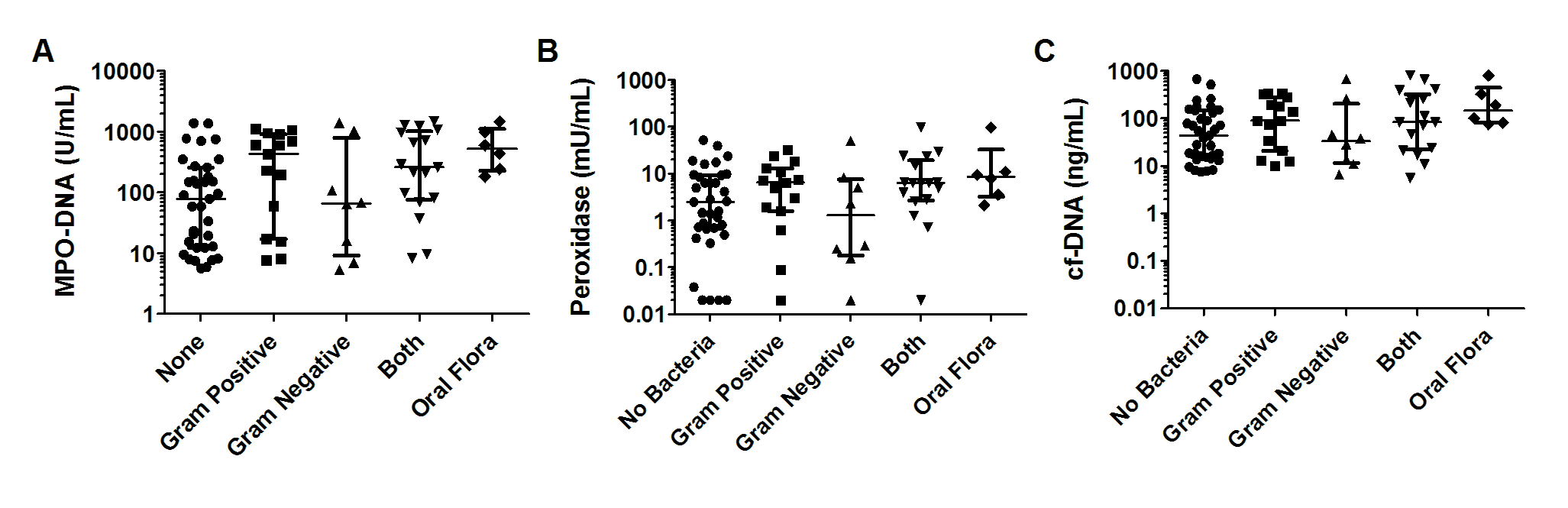
**

**NETs and bacterial type.** NETs are not associated with bacterial type. MPO-DNA, peroxidase, or cell-free DNA concentrations were compared amongst cultures with gram negative, gram positive, both forms of organisms and cultures that only grew oral flora. P values are for Kruskal-Wallis non-parametric ANOVA. Error bars show the median and interquartile range.
